# Psychiatric symptoms and disorders among Yazidi children and adolescents immediately after forced migration following ISIS attacks

**DOI:** 10.1007/s40211-016-0195-9

**Published:** 2016-09-15

**Authors:** Veysi Ceri, Zeliha Özlü-Erkilic, Ürün Özer, Murat Yalcin, Christian Popow, Türkan Akkaya-Kalayci

**Affiliations:** 1 Pendik Training and Research Hospital, Department of Child and Adolescent Psychiatry, Medical School of Marmara University, Fevzi Cakmak Mahallesi Mimar Sinan Caddesi No. 41, Üst Kaynarca, Pendik, Istanbul, Turkey; 2 Outpatient Clinic of Transcultural Psychiatry and Migration Induced Disorders in Childhood and Adolescence, Department of Child and Adolescent Psychiatry, Medical University of Vienna, Währinger Gürtel 18–20, 1090 Vienna, Austria; 3 Mazhar Osman Training and Research Hospital for Psychiatry and Neurology, Zuhuratbaba Mah.Dr.Tevfik Sağlam Cad. No:25/2, 34147 Bakırköy/İstanbul, Turkey; 4 Department of Child and Adolescent Psychiatry, Gazi Yasargil Education and Research Hospital of Diyarbakir, Üçkuyu Mh, 21010 Kayapınar/Diyarbakır, Turkey

**Keywords:** Children and adolescents, Refugees, Yazidis, Psychiatric symptoms and disorders, Mental health, Kinder und Jugendliche, Flüchtlinge, Jesiden, Psychiatrische Symptome und Störungen, Psychische Gesundheit

## Abstract

**Background:**

The aim of the present study was to evaluate psychiatric problems and disorders among Yazidi Kurd refugee children and adolescents, who were assessed immediately after their forced migration following life-threatening attacks by ISIS terrorists.

**Methods:**

We retrospectively analyzed the psychiatric assessments of 38 Yazidi children and adolescents (age 2–18, mean 12 years, m:f = 16:22), which were performed upon their arrival at the refugee camp.

**Results:**

All children and adolescents exhibited psychiatric problems and disorders, 50 % had one, and 50 % had more than one. The most relevant problems were disturbed sleeping (71 % of children), followed by depression (36.8 %), conversion disorders (28.9 %), adjustment (21.8 %), acute (18.4 %) and posttraumatic stress (PTSD, 10.5 %) disorders, and non-organic enuresis (18.4 %).

**Conclusion:**

Our study confirms the results of previous studies, asserting that refugee children and adolescents do not just suffer from PTSD but from various other problems that are already present in the first days of resettlement. Children and adolescents living in refugee camps urgently need psychosocial support.

## Zusammenfassung

**Hintergrund:**

Das Ziel der vorliegenden Studie war es, psychische Probleme und Störungen bei jesidisch-kurdischen Flüchtlingskindern und Jugendlichen, die unmittelbar nach erzwungener Migration aufgrund von lebensbedrohlichen Angriffen der IS-Terroristen untersucht wurden, zu erfassen.

**Methode:**

Die Autoren haben die psychiatrischen Diagnosen von 38 jesidischen Kindern und Jugendlichen (im Alter von 2 bis 18 Jahren, Altersdurchschnitt: 12 Jahre, 16 männlich, 22 weiblich), welche bei ihrer Ankunft im Flüchtlingslager durchgeführt wurden, retrospektiv analysiert.

**Ergebnisse:**

Alle Kinder und Jugendlichen zeigten psychische Probleme und Störungen, wobei 50 % eine und 50 % mehr als eine Störung zeigten. Die wichtigsten Probleme waren Durchschlafstörungen (71 % der Kinder), gefolgt von Depression (36,8 %), Konversionsstörungen (28,9 %), Anpassungsstörung (21,8 %), akute (18,4 %) und posttraumatische Belastungsstörung (PTBS, 10,5 %) und nichtorganische Enuresis (18,4 %).

**Schlussfolgerung:**

Unsere Studie bestätigt die Ergebnisse früherer Studien, dass Flüchtlingskinder und Jugendliche nicht nur an PTBS leiden, sondern auch an verschiedenen anderen Problemen, die bereits in den ersten Tagen ihrer Unterbringung präsent sind. Kinder und Jugendliche, die in den Flüchtlingslagern leben, brauchen dringend psychosoziale Unterstützung.

## Introduction

The Syrian civil war, now in its 5th year, has led to “the biggest humanitarian and refugee crisis of our time” [1]. Political protests against the Syrian regime increased from 2011 on. The reaction of the government against these protests evoked a brutal civil war, causing a gigantic wave of migration. More than ten million Syrians were forced to leave their homes, six million people migrating within Syria, four million leaving the country, a third of them women and minors. Turkey accommodated some two million refugees, among them a few thousand Yazidi Kurds, rescued from attacks of the Islamic State in Iraq and Syria (ISIS) in Northern Syria and Iraq.

Refugees, especially children and adolescents, exposed to or witnessing the cruelties of war are at extreme risk of developing physical and psychological illness [2]. Therefore, in addition to granting safe accommodation, refugees need physical and mental health care.

Traumatic experiences during the war, forced migration, not always being welcome in the host country, dramatic social change, and the threatening of one’s culture of origin are significant stress factors leading to individual physical and mental health problems [3–5]. Children, women, and elderly individuals are exceedingly susceptible to traumatic damage, and children with migration experience display more behavioural and emotional problems than children without traumatic experiences [3, 5–7], including anxiety disorders, depression, posttraumatic stress disorder (PTSD), difficulties in peer relationships, low self-esteem and dissatisfaction with life [8–20]. Forced migration and experienced violence may destructively influence the mental health of the individuals [4].

In August 2014, the terror organization ISIS attacked Yazidi Kurds living in Iraq/Sinjar (Şengal/Şingal), forcing them to leave their homeland and flee to safer places. More than 10,000 Yazidis sought refuge in Turkey including a large number of children. Yazidis reached Turkey in a harrowing march and were relocated in various camps in Eastern and Southeastern Anatolia. The Psychiatric Association of Turkey reported that about 10,000 Yazidi refugees are living in the camps of Diyarbakir, Cizre, and Silopi and the majority consist of children and women [21].

As there is not enough known about Yazidis, the authors find it useful to introduce some information about them. Yazidis are an ethnically Kurdish community, who mainly live in Nineveh province of Iraq, in Syria and Southeast Turkey, and speak *Kurmanji* a shared Kurdish dialect [22–24], Yazidism, a closed religion in regard to conversion of other people, is an ancient Mesopotamian religion which dates back to the Sumerian period [23]. As a result of misunderstanding by the followers of other religions Yazidism is the most oppressed religion in Iraq [23]. Yazidis are monotheists, who believe in one God “Xweda” (Ezda) the creator of the world, who sent seven angels to protect it. They believe that at first God created “Melek Taus”, the Peacock Angel, from his own illumination and six other angels later. They also believe that “Melek Taus”, who is the master of the other six angels, refused to bow to Adam. Because of this “Melek Taus” is often misunderstood to be Satan by other religious groups, as Satan also refused to bow to Adam. Thus Yazidis who respect and praise “Melek Taus” are wrongly called “devil worshippers”. Because of that they have been confronted with many genocides in their history, although Yazidis strictly refuse to kill others. Yazidis do not accept religious conversion and their children are baptized at birth. They are only endogamous and may not marry other Kurdish or other clans. Yazidi cultural narratives are based on oral tradition such as Strans (songs accompanied by music) and Kilams (songs with various themes). White is a holy colour for Yazidis. Also, the water of Kaniya Sipi “The White Spring”, is holy, especially during praying rituals and pilgrimage [25]. For Yazidis, earth, air, fire and water are also holy. They have many taboos to protect the purity of earth, air, fire and water. They believe that not following these taboos and traditions can lead to psychological and physical problems. And they also try to treat psychological and physical problems with different religious rituals [26].

We would also like to mention and explain some Yazidi idioms of distress. For Yazidis, the term “Ferman” is an expression for destruction and holocaust and reminds them of massacres against Yazidis; it means at once genocide and trauma. Every Yazidi knows the word “Ferman” because the term passes on from one generation to the next. In the context of the terror attacks by ISIS, the term “Ferman” regained a massive impact for Yazidis. It evokes feelings of mourning and fright. Two further idioms of distress are “nefsî” (arab. Psyche), which is used synonymously for all mental disorders and traumas, and “liver burned” (cigera min shewiti), which means emotional suffering [26].

We analyzed the available records of Yazidi refugee children, who were assessed upon arriving at Turkish refugee camps and sought medical and mental health support. The aim of the study was to describe psychiatric symptoms and disorders of a specific group of children who underwent forced migration and related traumatic experiences.

## Methods

Children and adolescents visiting the general health-care units in camps for a medical evaluation were assessed using a detailed psychiatric interview. Three of the children were admitted to the health-care units for psychological support. The other children and adolescents who showed psychiatric problems during the medical evaluation were advised to receive psychiatric care later.

All of the children were evaluated with a detailed psychiatric interview. The evaluations were made by the first author of this paper from September 10–19, 2014. The psychiatric diagnosis was made based on the evaluation of symptoms reported by parents and the psychiatric evaluation of children. The data collected from these assessments were retrospectively reevaluated by the same child psychiatrist.

The records consisted of 42 Yazidi refugee children, 24 girls, and 18 boys, aged between 2–18 years (mean 12.1 ± 4.5 (SD)), admitted to the refugee camps of Cizre, Silopi and Diyarbakir where about 3600 children are settled in these camps. The records of four children (two boys and two girls) were not complete and therefore excluded from the analysis. All children were members of large families with a mean of 6.8 siblings.

Psychiatric diagnoses were mainly assigned according to DSM-5 criteria [27]. Statistical calculations, mainly descriptive analyses, were performed using SPSS 16.0 software [28].

## Results

The assessed children and adolescents reported that they had to walk for days without eating, drinking or sleeping. They were very afraid of being captured by ISIS or not to wake up again after sleeping, even when they were already staying in the camp. They also reported they did not feel safe in the camp at all, and they did not understand why they were involved in the war and threatened with death. They told that they could not trust anybody and that they did not want to return to their home. They did not discuss these fears with their families, but shared their thoughts with their peers. Several children reported that they had witnessed some parents leaving their dead children behind while being on the run.

During the first few days most children were shy and avoided contact, but thereafter they sought contact with the custodians and continuously tried to attract their attention. They also preferred to stay and play alone and tended to avoid contact with their peer group.

Nine of the assessed children were forcibly separated from their first-degree relatives. Seven of these children knew that their close relatives were alive, and could contact them by telephone, whereas two children could not get any information about their fathers. One of the children reported that his father was killed by ISIS.

None of the assessed children witnessed a homicide or a violent situation. Most of them reported that they followed acts of ISIS through social media, including beheading and bombing. Almost all of the 32 children stated that they have heard how ISIS captures and retains girls to be sold, killed, wedded, or raped.

The majority of the assessed children (71 %) reported sleeping problems, such as problems falling asleep, frequent awakenings, and parasomnias like somnambulism and nightmares.

Table 1 gives an overview of the recorded complaints that were mentioned by the caregiver of children and adolescents.

**Table 1:** Symptoms of the refugee children and adolescents (*n* = 38)

Reason of referral	*n*	%
Sleeping problems	27	71.1
Impaired social inclusion	15	39.4
Somatic complaints	14	36.8
Fainting	12	31.5
Irritability	12	31.5
Anxiety	11	28.9
Enuresis	9	23.7
Intrusive thoughts about death	6	15.8
Eating problems	5	13.1

Table 2 gives an overview on the predominant psychiatric diagnoses. The most common diagnosis was depressive disorder. More than one of three children was diagnosed to have depressive disorder.

**Table 2:** Psychiatric diagnoses of the children and adolescents

Diagnoses	*n*	%
Major depressive disorder	14	36.8
Conversion disorder	11	28.9
Adaptation disorder	8	21.8
Acute stress disorder	7	18.4
Enuresis	7	18.4
Posttraumatic stress disorder	4	10.5
Separation anxiety disorder	4	10.5
Somatization disorder	3	7.8
Selective mutism	1	2.5
Night terrors	1	2.5

## Discussion

Analyzing the available psychiatric records of Yazidi Kurd refugee children in Turkish detention camps immediately following forced migration, we found most children severely traumatized although they had not witnessed violent acts. They mostly suffered from sleeping, stress related, and anxiety disorders, depression, and somatic complaints like non-organic enuresis, and they reacted withdrawn and fearful. Overall, 50 % were assigned one, the other 50 % two or more psychiatric diagnoses. Although the number of assessed children and adolescents is low—probably due to a systematic “neglect” of symptoms—we consider the reported symptoms to be the tip of the iceberg, representing a much larger, undetected problem.

Our results are concordant with similar investigations in children who were war-affected or experienced political violence, or forceful migration in Kosovo [29, 30], Uganda [13], Somalia [31], Darfour [32, 33], East Congo [18], Sudan [20], Cambodia [34], Bosnia [30, 35], Syria [15], and others [11, 36]. In addition to exacerbations of pre-existing problems [15], novel psychopathologies were observed, mainly related to sequelae of anxiety and depression. All these problems strongly impede the quality of life of the children for a prolonged period after escaping from the worst.

The most prevalent observed mental health problem (71 %) was sleep disturbance—partly related to the high levels of stress experienced on the run and thereafter, and to the problems related to depression and anxiety disorders. Disturbed sleep will aggravate the high-arousal emotional state, the development of posttraumatic symptomatology in a vicious circle, and delay or impede recovering [37].

Dissociative disorders (8 % in our sample) were also frequently observed and may be understood as an immediate defence reaction to traumatic experiences, an involuntary escape from reality. Because recognizing dissociative symptoms in children may be difficult [38], an even higher prevalence in our study may be suspected. Detecting dissociative disorders is essential because treatment is indispensable.

Our refugees were in stage 2 of the three-stage process of migration [39], stage 1: considering migration, stage 2: arrival and stay at a detention camp, stage 3: integration into the host country. Therefore, PTSD was only diagnosed in a minority of about 10 %. Studies performed later in the process (stage 3), showed a much larger prevalence of 31–55 % [40, 41]. This difference may be explained by the time lag that exists between the traumatic event(s) and the onset of PTSD symptoms [42].

The evident underutilization of mental health services by refugee children—even if available—is well documented among traumatized patients [43] and is probably related to shame of complaining, to coping strategies, or to the “tough” climate in the refugee camp. Jones et al. [29] described that children witnessing war cruelties in Bosnia impressed the medical staff by their seemingly hard-boiled attitude, taking large emotional distance to gruesome events. Furthermore, accompanying adults often are not aware of the posttraumatic internal processes in the children—documented, e. g. by the low correlations between information given by parents, teachers or guardians and the patients [2]: self-perceived vs. informant severe psychological distress in 50 vs. 33 (guardians) and 36 % (teachers) [2], either because the children hide their psychological wounds or because relatives and carers became “hard-boiled” themselves in order to cope with the horror or because of being grateful for having survived. The problems of continued “neglecting” of the effects of trauma, insufficient environmental support and sometimes continued threatening [17] leads to continued distress, deleterious effects of undertreatment [40, 44] and aggravates the development of PTSD [45, 46].

In various camps in Eastern and Southeastern Anatolia where Yazidi Kurds were relocated, there are unfortunately just limited treatment possibilities in case of psychological problems. In general health-care units of camps, a general practitioner and nurse from the ministry of health work regularly by day. Additionally, volunteer adult psychiatrists and child psychiatrists examine and treat refugees by day between 17:00 and 20:00. The first author of this paper worked also voluntarily as a child psychiatrist in these camps.

One of the strengths of our paper is the fact that the children were assessed in their mother tongue by an experienced child psychiatrist. The majority of studies conducted in refugees are either based on self-report instruments or on interviews held with the support of an interpreter, leading to distorted results [40].

Assigning psychiatric diagnoses to traumatized children will help identifying their therapeutic needs. This seems important because delays in treatment will aggravate the risk of more severe clinical pictures and long-term physical and psychic impairment [47]. Unfortunately, only a few refugee programs provide psychosocial support [44].

## Limitations

Our study bears a number of limitations: the psychiatric interviews for many reasons were only performed in the few children claiming medical or psychiatric support. Therefore our study sample may not represent the majority of the refugees. Larger studies in migrant children (e. g. [2]) showed about half of the refugees were affected with psychiatric problems. Second, the psychiatric diagnosis was assigned using the information gathered from family members and interview of the children. We have no other source of information, e. g. teachers, guardians, peers, other refugees. This may have introduced an informant bias. Third, the cultural differences in expression of excessive stress make it difficult to assign a straight psychiatric diagnosis.

The major strength of our study is the immediate and careful assessment of psychiatric symptoms and disorders by an experienced Kurdish child psychiatrist, who was not at the mercy of help from interpreters or self-report questionnaires.

## Conclusion

Forced migration causes multiple traumatic and posttraumatic experiences that lead to severe, often undetected psychiatric symptomatology and disorders. Knowing about the high risk of developing a multitude of psychiatric disorders that require treatment and the mechanisms and consequences of neglecting symptomatology, should help in procuring adequate medical and psychotherapeutic support. Further studies systematically assessing all possibly traumatized refugee children will provide a better estimate of the prevalence of psychiatric disorders in this population. A safe environment, enduring well-organized support and professional treatment including the families will be a way to help the poorest, who lost their livelihood.
